# Volvox and volvocine green algae

**DOI:** 10.1186/s13227-020-00158-7

**Published:** 2020-07-01

**Authors:** James G. Umen

**Affiliations:** Donald Danforth Plant Science Center, 975 N. Warson Rd, St. Louis, MO 63132 USA

**Keywords:** *Volvox carteri*, *Chlamydomonas reinhardtii*, Multicellularity, Green algae, Morphogenesis, Comparative genomics

## Abstract

The transition of life from single cells to more complex multicellular forms has occurred at least two dozen times among eukaryotes and is one of the major evolutionary transitions, but the early steps that enabled multicellular life to evolve and thrive remain poorly understood. Volvocine green algae are a taxonomic group that is uniquely suited to investigating the step-wise acquisition of multicellular organization. The multicellular volvocine species *Volvox carteri* exhibits many hallmarks of complex multicellularity including complete germ–soma division of labor, asymmetric cell divisions, coordinated tissue-level morphogenesis, and dimorphic sexes—none of which have obvious analogs in its closest unicellular relative, the model alga *Chlamydomonas reinhardtii*. Here, I summarize some of the key questions and areas of study that are being addressed with *Volvox carteri* and how increasing genomic information and methodologies for volvocine algae are opening up the entire group as an integrated experimental system for exploring the evolution of multicellularity and more.

## Natural habitat and lifecycle

*Volvox* is a polyphyletic genus of multicellular freshwater green algae (Chlorophyta) that belong to a larger taxonomic grouping within the Order Chlamydomonadales known as volvocine algae [1]. The species *Volvox carteri* f. *nagariensis* (hereafter *V. carteri* or Volvox unless otherwise specified) is the best-studied species in the genus, and has become an established model for developmental and evolutionary biology [2]. Two other *V. carteri* forma that are available in culture collections, f. *kawasakiensis* and f. *weismannia*, are closely related but different species from *V. carteri* f. *nagariensis.* Volvocine genera are cosmopolitan and have been collected on all continents except Antarctica [3, 4]. The habitats of *V. carteri* include small bodies of water (ponds, rice paddies) and larger lakes/reservoirs [3].

**Fig. 1:**
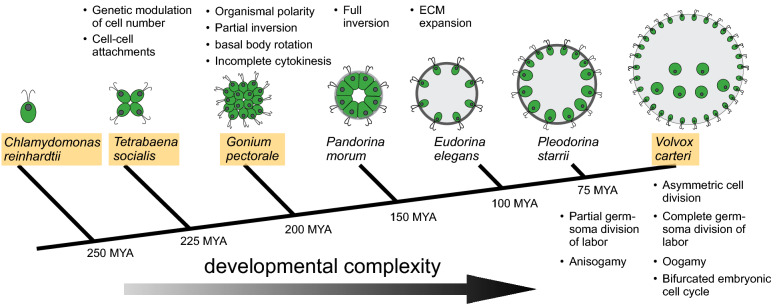
Volvox and volvocine algae. Simplified cladogram of selected volvocine species from representative genera shown in cartoon form with successive cellular and developmental innovations indicated by bulleted descriptions above or below the node in which they arose. Volvocine algal cells are polarized with an apical pair of flagella just above the nucleus and a single large chloroplast located basally. Species with published sequenced genomes have names in tan boxes. Light grey shading depicts extra cellular matrix (ECM) that occupies increasing amounts of spheroid volume in successive genera. Estimated divergence times at each node are based on data in [6]. This figure was adapted from [92] and reproduced here under Creative Commons Licence CC BY 4.0

While on its own Volvox is a highly tractable model organism, it has even greater utility when it is studied in the context of the volvocine algal clade which includes several additional multicellular genera with different sizes, cell numbers and degrees of developmental complexity (e.g., *Tetrabaena*, *Gonium*, *Astrephomene*, *Pandorina*, *Yamagishiella*, *Eudorina*, *Pleodorina*), and a close unicellular outgroup species, the model alga *Chlamydomonas reinhardtii* (Chlamydomonas) [2, 5] (Fig. 1). The divergence time between the common ancestor of *C. reinhardtii* and multicellular volvocine species is estimated at around 250 MY [6], though the fossil record for green algae is sparse and complicated by homoplasy that makes definitive taxonomic affiliation and time calibration difficult [7]. No other group of extant organisms captures the crucial transition of eukaryotic life from unicellular to multicellular as lucidly as the volvocine clade, and much of modern Volvox research is motivated by the potential to reconstruct the early stages of this transition in unprecedented detail [1, 8].

**Fig. 2:**
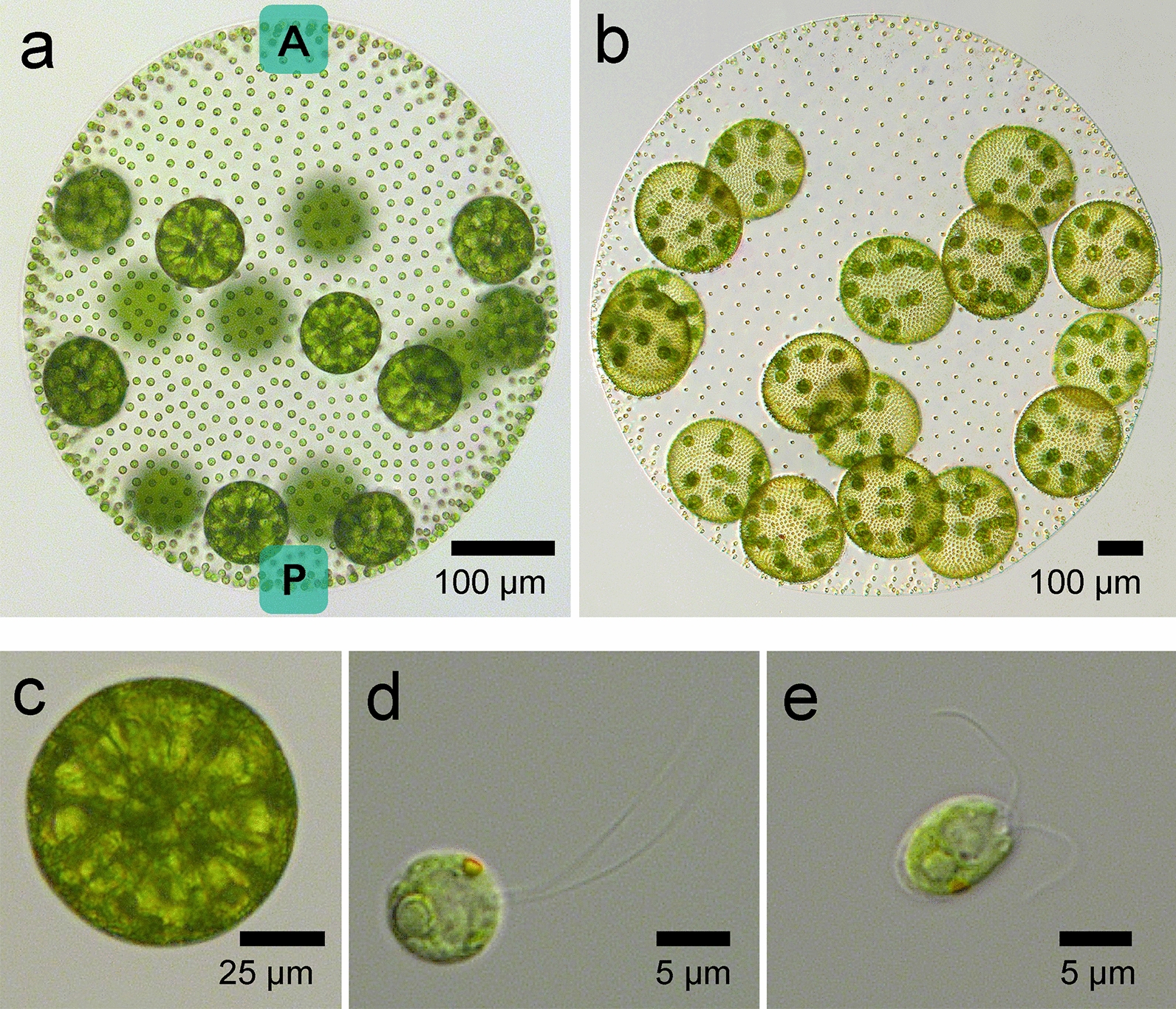
*Volvox carteri.***a**–**d** Light micrographs of vegetative phase *Volvox carteri* (Volvox) showing a mature pre-cleavage stage adult with the anterior (A) and posterior (P) poles labeled (**a**); a mother spheroid with juveniles (**b**); an isolated gonidium (**c**); a somatic cell from a mature pre-cleavage adult spheroid (**d**). The orange/brown-colored eyespot on the somatic cell is visible near the periphery at around 1 o'clock, and the parallel-oriented flagella are visible near 2 o'clock.  **e**. Light micrograph of a *Chlamydomonas reinhardtii* cell with flagella oriented to beat in opposite directions. This figure was adapted from [92] and reproduced here under Creative Commons Licence CC BY 4.0

Vegetative phase *V. carteri* are spheroids of several hundred microns diameter which are organized in a simple radially symmetric multicellular body plan with just two cell types: ~ 2000 small sterile biflagellate somatic cells that are similar in size to a Chlamydomonas cell, and 12–16 large aflagellate reproductive cells called gonidia (Fig. 2a–e). The somatic cells are positioned around the exterior of the spheroid and provide motility, while the gonidia are distributed in the posterior region just below the surface. All of the cells are embedded within a clear secreted glycoprotein-rich extracellular matrix (ECM) that occupies around 99% of the spheroid volume.

**Fig. 3:**
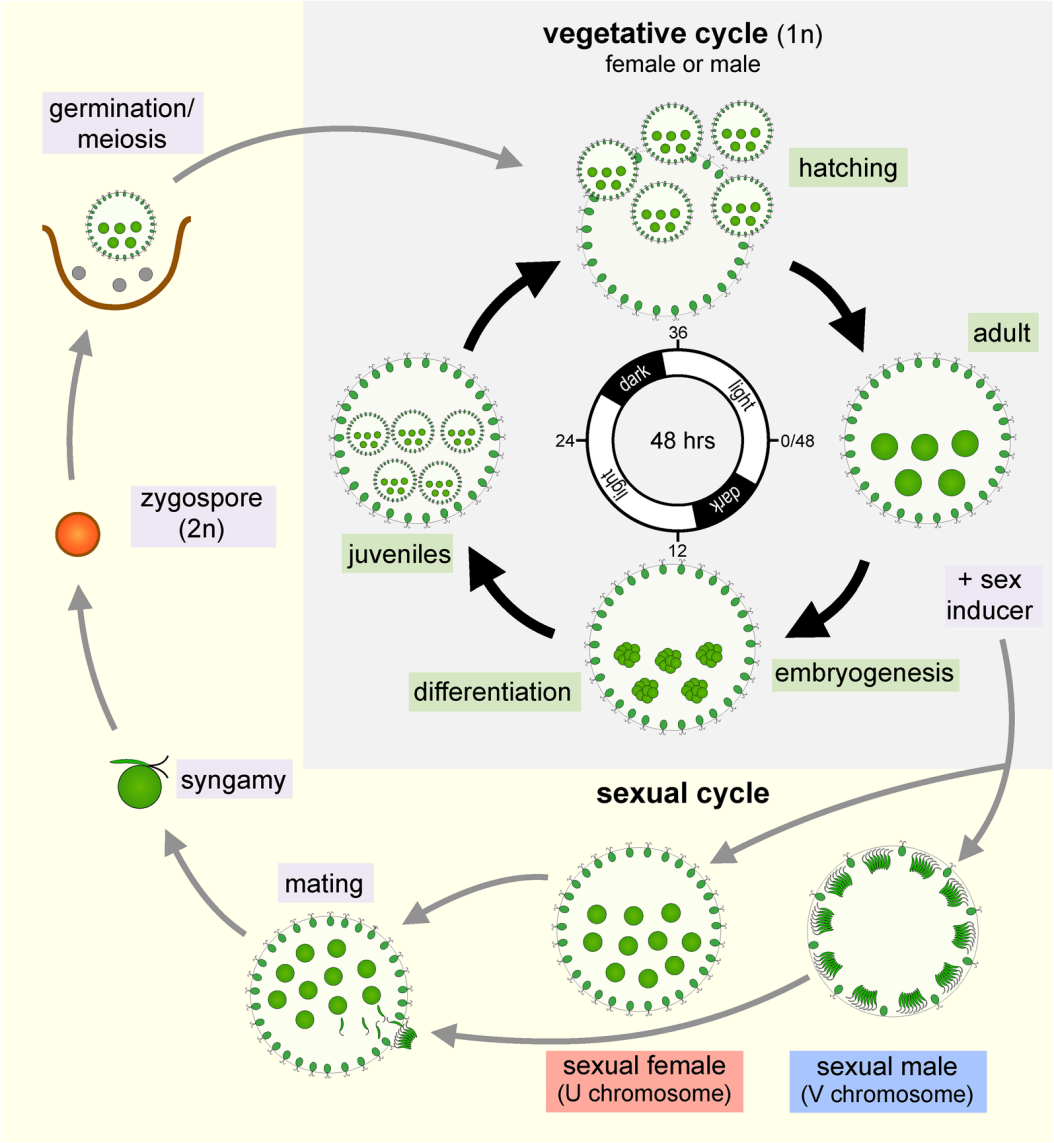
Volvox life cycle. Schematic of the Volvox vegetative and sexual life cycle phases. Vegetative reproduction (grey shaded region) occurs in the haploid (1n) phase and can be synchronized with a 16 h:8 h light:dark diurnal regime (center of diagram), under which one full reproductive cycle is completed every 48 h. Vegetative development starts with mature pre-cleavage adults (~ 3:00 on diagram, corresponding to spheroid in Fig. 2a) and proceeds clock-wise through embryogenesis and then cyto-differentiation of germ cells (gonidia) and somatic cells to form juveniles (~ 9:00 on diagram, corresponding to spheroid in Fig. 2b), hatching of juveniles, and finally maturation to complete the cycle as the next generation of adults. After hatching, the parental somatic cells of the previous generation are discarded and undergo senescence and cell death. Sexual development is triggered by exposure to sex inducer and leads to gonidia undergoing modified embryogenesis and development (not shown) into adult sexual egg-bearing females or adult sexual sperm-packet-bearing males. Sperm packets are released and swim to females where mating takes place with internal fertilization (syngamy), resulting in formation of diploid zygospores. Meiosis occurs upon germination and produces three polar bodies and one haploid progeny that re-enters the vegetative life cycle. This figure was adapted from [10], and reproduced here with permission from the *Annual Review of Microbiology*, Vol. 73 © 2019 by Annual Reviews, http://www.annualreviews.org

Volvox and all volvocine species have a haplontic life cycle (Fig. 3) where vegetative (i.e., mitotic) haploid-phase reproduction can be maintained indefinitely, and where sex is triggered by either nutrient or hormonal signals. Nitrogen deficiency is the cue for sexual differentiation in most volvocine genera, but in Volvox, a species-specific glycoprotein hormone called sex inducer (SI) is the trigger for sex. SI production can be induced by heat shock [9], and when vegetative gonidia are exposed to SI they undergo modified embryogenesis leading to development of sexually dimorphic males or females under the control of a haploid UV sex chromosome system [10–12]. Fertilization produces diploid zygotes which mature into thick-walled dormant zygospores. Zygospores are environmentally resistant and can remain viable for years in a frozen or desiccated state. Exposure of zygospores to light and nutrients initiates germination and meiosis to produce recombinant haploid progeny that re-enter the vegetative life cycle [13]. Detailed descriptions of mating and fertilization can be found elsewhere [3, 14].

## Lab culture and field collection

Field samples of Volvox are routinely collected in one of two different ways: Starr and colleagues described a simple means of germinating zygospores from dried soil samples that are usually collected near bodies of water where Volvox has been observed [14]. Dried soil is advantageous as it can be transported and stored easily, though successful germination is never guaranteed. The alternative is collection of live samples using a phytoplankton sieve as demonstrated in this video from Dr. Hisayoshi Nozaki’s field studies [15].

**Fig. 4:**
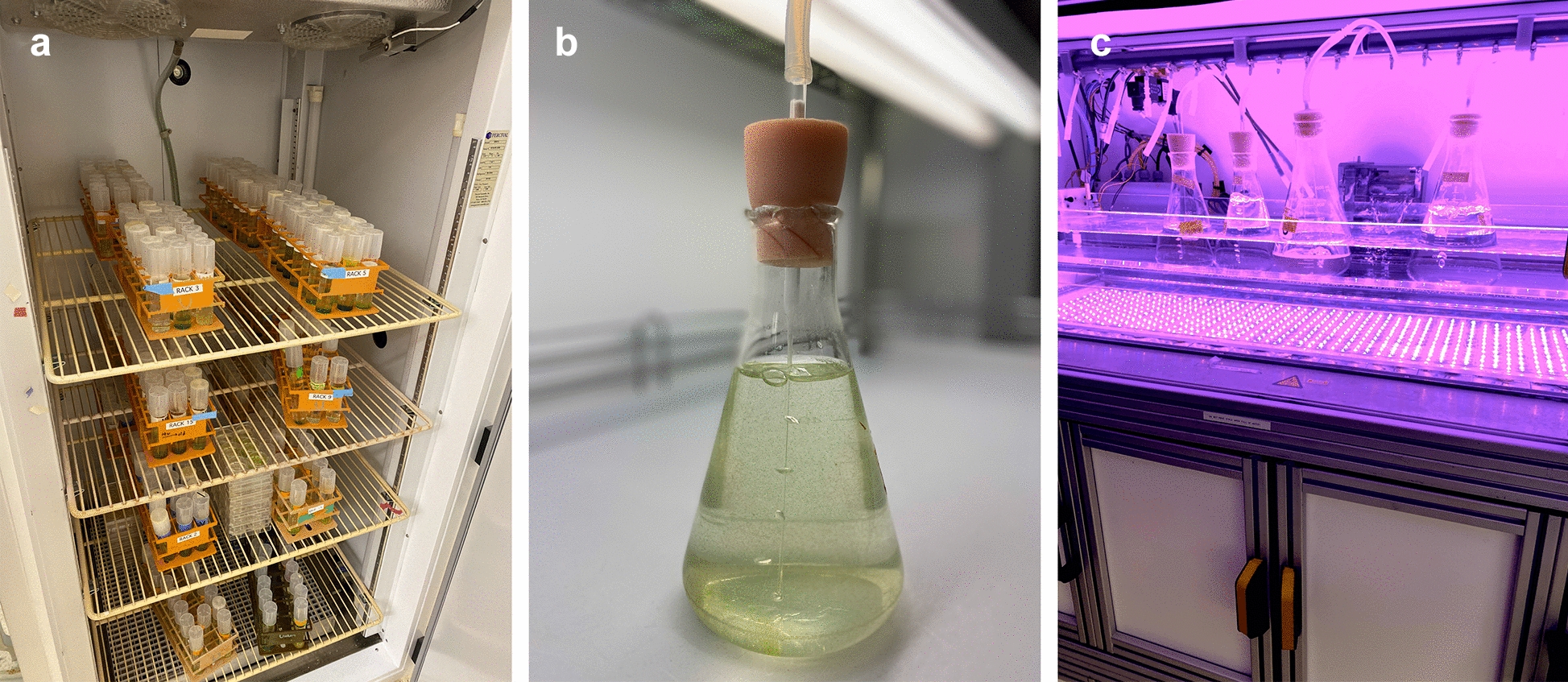
Volvox cultures. **a** Stock cultures kept in temperature- and light-controlled growth chamber. **b** Volvox culture in 500-mL flask plugged with silicone sponge stopper and aerated via cotton-plugged Pasteur pipette. Individual spheroids are just barely visible to the naked eye. **c** LED growth chamber for synchronizing cultures. Culture flasks similar to that in panel **b** sit in a temperature-controlled acrylic water bath and are lit from below by red and blue LEDs that can be programmed for a diurnal regime. A gas manifold above the flasks provides pressurized air for culture aeration

A single male–female pair HK9 and HK10 (a.k.a. Adm and Eve) germinated from soil collected near Kobe, Japan, are founders for standard strains still used by most laboratories [3, 14]. The HK9 male strain acquired a morphological mutation and was replaced with a normal male progeny (69-1b) derived from a cross with HK10 [16]. Additional strains, including newer isolates [17, 18], are available from several culture collections (see below). For routine studies female strains (e.g., Eve) are preferred, as males can spontaneously undergo sexual differentiation—an irreversible developmental transition for males whose sperm and somatic cells cannot de-differentiate [3]. Because sexual males produce additional SI, a single spontaneous sexual male arising in a culture can sexually induce the entire population of males, leading to its extinction. Cultures are usually maintained in defined liquid media such as Standard Volvox Medium (SVM) [19] in a temperature- and light-controlled environment, i.e., a plant growth chamber or similar setup (Fig. 4a). Optimal growth is at 32C under moderately strong light (200–300 µE m-2 s-1 white light, 16 h:8 h L:D cycle), with aeration provided by bubbling (Fig. 4b), and under these or similar conditions the vegetative life cycle can be synchronized, with a new generation produced every 48 h (Fig. 3). Small-scale stock cultures (20-40 mLs) in glass tubes can be grown without aeration and maintained at room temperature (~ 25C) on light shelves or in incubators (Fig. 4a). Maintaining cultures axenically is ideal and requires meticulous sterile technique, but methods exist to remove microbial contaminants. Although there is one report of successful cryopreservation of Volvox [20], maintenance of Volvox cultures generally requires live passaging every 2 to 4 weeks. Dried or frozen zygospores are an alternative method for long-term storage, but this approach cannot maintain a completely isogenic strain background.

## Major interest and research questions

Volvox is a rich developmental system where the origins of developmental complexity and innovation can be inferred using comparative methods and direct experimental testing [1, 5, 21, 22]. The late David Kirk, whose laboratory played a major role in ushering Volvox research into the modern molecular era, summarized 12 key steps or innovations in the progression from a unicellular Chlamydomonas-like ancestor to *V. carteri* [21] (summarized in Fig. 1, with subsequent refinement/elaboration by others [5, 23, 24]). The earliest innovations include cell adhesion, genetic control of cell number, acquisition of organismal polarity, incomplete cytokinesis with formation of cytoplasmic bridges between embryonic blastomeres, morphogenetic shape changes (a precursor to the process of inversion described below), rotation of basal bodies to enable coordinated flagellar motility, and conversion of the cell wall to extracellular matrix (ECM) and colony boundary. Subsequent innovations included expansion of the ECM that enabled rapid organismal enlargement, partial and complete germ–soma division of labor, asymmetric cell divisions, bifurcated embryonic cell division programs, and anisogamy/oogamy. Deciphering the molecular genetic origins of these steps continues to be an exciting and increasingly approachable goal. A sampling of these innovations and related topics are discussed below in reference to Volvox.

### Germ–soma cell-type specialization

Terminally differentiated somatic cells are a hallmark of complex multicellular organisms and are likely to have arisen independently at least three times in volvocine algae [5]. In *V. carteri*, maintenance of somatic differentiation is governed by a somatic cell-specific SAND domain transcription factor, RegA, which is part of a volvocine algal specific VARL family that underwent a complex history of duplications in multicellular volvocine genera [25–27]. Germ–soma differentiation in V. carteri occurs shortly after embryogenesis is complete, and is dictated by postembryonic cell size; but the coupling between cell size and cell fate remains a mystery [28]. Recent cell-type transcriptome studies in Volvox have also shed light on how germ and somatic cells differ, and the potential origins of their specialized differentiation programs [29–31]. Much remains to be discovered in this system including identifying direct targets of RegA, identification of germ cell-specific differentiation factors, and determining how asymmetric cell divisions governed by the chromatin-associated chaperone proteins glsA, Hsp70A and other factors are specified and executed [32].

### Embryogenesis and morphogenesis

Volvox has evolved some remarkable developmental innovations which have no clear analogs in its unicellular relative, Chlamydomonas. These include asymmetric embryonic cell divisions and a bifurcated embryonic cell division program where both the number of cell divisions and their symmetry differ in a predictable manner among cleaving blastomeres. This developmental bifurcation gives rise to a post-cleavage embryo containing ~ 2000 small somatic-precursor cells arranged like a hollow-ball with 12–16 exterior-facing large gonidial-precursor cells in predictable evenly spaced locations around the embryo’s anterior region. The remarkable process of inversion immediately follows embryonic cleavage and involves a sequence of coordinated cell shape changes that enable the embryo to turn itself completely inside-out and assume its adult configuration with outward-oriented somatic precursors and interiorly located gonidial precursors [33]. Anterior–posterior (A-P) embryonic polarity and the presence of cytoplasmic bridges connecting post-mitotic blastomeres are key derived features that enable inversion to occur [34–37]. Mutants that disrupt inversion and other developmental programming have been isolated and hold great potential for understanding the origin of these innovations (reviewed in [2]).

### Evolution of sexes and sex chromosomes

As described above, Volvox has separate male and female sexes whose differentiation is controlled by a pair of haploid UV sex chromosomes [12] that evolved from an ancestral mating-type locus [10, 11] The entire volvocine lineage has been used as a test case for predictions about how/why anisogamy and oogamy evolved in response to an increase in organismal size and cell number [10, 24]. The conserved RWP-RK family transcription factor, Mid (*minus* dominance) resides on the male chromosome (or *minus* mating-type locus in isogamous species) and controls male or *minus* sexual differentiation in dioicous species of volvocine algae [10]. *Plus* mating type or female are the default differentiation programs when Mid is absent. Monoicy (i.e., epigenetic sex determination) is present in some species, but dioicy is likely to be ancestral. In Volvox Mid is inferred to have evolved control of more complex developmental programming than in isogamous volvocine species so that it can promote male sexual differentiation and sperm development [38, 39]. Although Mid controls key aspects of Volvox sexual germ cell differentiation, other genes on the Volvox UV sex chromosomes play additional roles in specifying some aspects of male versus female embryonic patterning and in promoting sex-specific reproductive fitness. This finding and others fit predictions of evolutionary theory [40] where sex chromosomes are postulated to acquire genes required for reproductive fitness of males or females. Other derived aspects of the Volvox sexual cycle such as SI-triggered signal transduction, internal fertilization, and reduced meiotic products are fascinating and relatively unexplored areas of interest [10, 24].

### Genome evolution/comparative genomics

The Chlamydomonas nuclear genome sequence was published in 2007 [41], and the Volvox genome followed in 2010 [1, 42]. Two other volvocine nuclear genomes for the multicellular genera, *Gonium* and *Tetrabaena*, have also been published [43, 44], with more likely to follow soon. Additional genomic resources including some mating loci sequences, unannotated nuclear genome assemblies, and selected genomic regions are also available but have no public browsers or search engines [11, 25]. By most metrics volvocine genomes do not differ significantly in size or protein coding capacity, a finding which suggests that developmental innovation occurred mostly through modification or repurposing of a common ancestral genetic “toolkit” [42]. Noncoding genomic regions among the four sequenced species show high divergence, limiting their potential for predicting cis-regulatory elements based on sequence conservation. Additional regulatory complexity derived from microRNAs or small non-coding RNAs may also exist in Chlamydomonas and Volvox, but little is known about how these pathways are involved in basic cellular processes or development [31, 45–47].

### Motility

Motility in Volvox and other volvocine species poses interesting developmental, evolutionary and biophysical questions [48–50]. Volvox swims while rotating about its A-P axis which is oriented in the direction of swimming (Fig. 2). To achieve coordinated swimming and phototaxis, somatic cells must be oriented correctly along the A-P axis, with their two flagella beating in parallel—a configuration that is achieved through each basal body rotating 90° about its long axis from a starting 180° orientation present in newly formed somatic cells. The 180° basal body orientation is an ancestral configuration that was retained in unicellular relatives such as Chlamydomonas which swims using a breast-stroke motion (Fig. 2d and 2e)[51, 52]. Flagellar responses to light in each somatic cell are mediated by an eyespot—an organelle containing channel rhodopsin photoreceptors which are shielded from light on one side by carotenoid-rich pigment granules that visibly mark the eyespot as a brown/orange dot on the side of the cell (Fig. 2d). This  configuration enables the channel rhodopsin in each eyespot to act as directional light sensor whose open/closed state transitions are tuned to allow synchronization between swimming rotation frequency and flagella beating responses mediated by calcium signaling. Together, the eyespots act as a distributed sensing system allowing the flagella of somatic cells on the side of the spheroid rotating past the path of incoming light to transiently adjust their beat-stroke and effect a turn towards or away from the light [53–55].

## Experimental approaches

Volvox is amenable to many types of experimental manipulations and methodologies. Because it can be easily synchronized using a diurnal regime, uniform populations from different developmental or life cycle stages can be obtained. At many (but not all) stages the germ and somatic cells (or cleaving embryos) can be physically separated and purified, thus enabling studies of individual cell types [29, 30]. Pre-cleavage or cleaving embryos can be released from parental spheroids. They remain viable and can complete development, allowing them to be studied in detail using time lapse imaging, or can be subject to various experimental manipulations [56]. Volvox is amenable to most standard methods of microscopic imaging including single plane illumination microscopy (SPIM, a.k.a. light sheet microscopy) to study development and morphogenesis [33, 57], and video microscopy to study motility and flagella dynamics. A recent study was able to establish in vitro motility in “zombie” Volvox whose cells were permeabilized but whose flagella could be re-activated in the presence of ATP and calcium to investigate flagella responses to calcium signaling [54].

Molecular genetics methods for Volvox make it a highly tractable system for developmental genetics. Classical genetics played a critical role in defining different classes of developmental mutants [58–62], but was superseded by the more facile method of transposon tagging which was the method of choice prior to having a genome sequence [35, 63–65]. With a sequenced genome, isolates that have abundant sequence polymorphisms [66], and low-cost resequencing methods, the prospects for using classical developmental genetics are again excellent. It should be noted that strains tend to lose sexuality during repeated vegetative propagation, but this can be remedied by routine crossing to maintain sexual fitness.

Volvox can be transformed using biolistic methods (DNA-coated gold particles delivered with a helium gene gun) coupled with any of several available selection markers [67–71]. Nuclear transgenes are thought to integrate randomly as they do in Chlamydomonas, and can be subject to silencing and position effects (i.e., influence on expression by nearby endogenous loci) [72]. During selection the spheroids grow for one or more generations, with non-transformed individuals eventually dying, and stably transformed genetically homogenous transformants emerging. Although overall transformation frequency is not high (typically several to a few dozen per biolistic shot targeting ~ 30,000 cleaving embryos, each with up to 16 germline precursor cells), co-transformation efficiency is quite high (typically 20–80%) making it relatively straight forward to express a gene of interest when it is co-bombarded with a selectable marker gene [70]. Regulated promoters have also been described [73, 74]. RNAi-mediated knockdowns using antisense [75, 76] or hairpin constructs [39] have been successful for reverse genetics and will likely remain useful for targeting essential genes where a knockout might be lethal, but will likely be replaced by CRISPR–Cas9 (or similar) genome editing that was successfully adapted for Volvox [77].

Additional methods or advances that would positively impact Volvox research include a reliable method for cryopreservation, more efficient transformation methods, a means of inserting transgenes at defined locations, and non-endogenous regulated promoters/transcription factors that can be controlled with small molecules or other non-physiological stimuli. Adapting CRISPR–Cas9-based genome editing to make targeted mutations was a huge breakthrough [77], and the added ability to do more directed and defined genome editing (e.g., creating site specific mutations, adding epitope tags, or creating fusion proteins transcribed from endogenous loci) would open up additional avenues of experimentation.

While *Volvox carteri* and *Chlamydomonas reinhardtii* have well-established molecular genetic methods, most other volvocine algae have few or no tools, with successful transformation reported for a handful of other species [78–81]. With more interest and improved genomics resources catalyzed by inexpensive genome and transcriptome sequencing, these current limitations can be overcome and will help further elevate the entire clade as a premier model for comparative and mechanistic studies of evolution and development.

## Research community and resources

Around a dozen laboratories worldwide use Volvox or its multicellular relatives, while the Chlamydomonas community is considerably larger. A biennial International Volvox Conference is held in odd years, with the most recent one in Tokyo, Japan in 2019 [82]. The Volvox meeting is staggered with a biennial International Chlamydomonas meeting held in even years in which Volvox and volvocine algae research are included [83]. Phytozome [84, 85] hosts data for the *V. carteri* and *C. reinhardtii* genomes and has some useful browser capabilities for viewing external data. Phycosm [86] hosts the former two species plus *Gonium pectorale*, while the *Tetrabaena socialis* genome assembly is not currently hosted in the public domain [44]. As mentioned above, there are some additional genomes or parts of genomes available, but with no browsers and/or limited annotations available [11, 25]. Key culture collections for obtaining Volvox and volvocine algal strains and/or media recipes are UTEX in the US [87], NIES in Japan [88] and SAG in Germany [89]. Excellent video microscopy of Volvox and its relatives can be found online [90] from Jeremy Pickett-Heaps and colleagues. A short list of recommended reading for learning more about different aspects of Volvox biology includes these references: [2, 3, 8, 10, 48, 49, 91].

## Data Availability

Not applicable.

## Abbreviations

Hr: Hour
ECM: Extracellular matrix
SI: Sex inducer
A-P: Anterior–posterior
